# An unusual foreign body as cause of chronic sinusitis: a case report

**DOI:** 10.1186/1752-1947-4-157

**Published:** 2010-05-26

**Authors:** Theodoros Kelesidis, Sara Osman, Harry Dinerman

**Affiliations:** 1 Department of Medicine, Caritas St Elizabeth's Medical Center, Tufts University School of Medicine, Boston, MA, USA

## Abstract

**Introduction:**

The presence of a foreign body in the nose is a relatively uncommon occurrence. Many unusual foreign bodies in the nose have been reported in the literature, but no case of a nasal packing occurring as a foreign body in the nasal cavity for a prolonged time has been found.

**Case presentation:**

We describe a unique case of the largest foreign body left *in situ *in the nasal cavity for over 10 years. Our patient was a 71-year-old Caucasian man with diabetes. Because of this, he was at high risk of developing complications from the foreign body and the chronic sinusitis. Amazingly, though, the foreign body had not caused any symptoms on our patient for many years, except for nasal discharge during the last few years. To the best of our knowledge, this is the first case in the literature of such a large intra-nasal foreign body described in an adult without mental illness and without trauma that remained *in situ *for such a long time.

**Conclusion:**

Undoubtedly, even illnesses with no complications could prove difficult for clinicians to diagnose. Clinicians should recognize the underlying causes that are responsible for the symptoms of chronic sinusitis and a unilateral nasal discharge should be assumed to be caused by an intra-nasal foreign body until proven otherwise.

## Introduction

The presence of a foreign body in the nose is a relatively uncommon occurrence. Unlike foreign bodies in other parts of the body that often produce noticeable symptoms, foreign bodies in the nose can go unrecognized for significant periods of time. A prolonged period of impaction is even less common, but it is more likely when the foreign body is an inert object. Many unusual foreign bodies have been reported in the literature, but no case has been found of a nasal packing occurring as a foreign body in the nasal cavity for a prolonged time.

## Case report

We describe a case of a 71-year-old Caucasian man with history of underlying cardiomyopathy and type 2 diabetes mellitus for 20 years. He also had a history of multiple hospitalizations for congestive heart failure. Our patient presented to us with worsening leg edema and weight gain. He had no fever, headache and denied other symptoms. However, he was noticed to have a foul smelling discharge from the right nostril. Upon further assessment, he mentioned that he had this symptom for years, but never complained about this and he never had a work-up for this nasal discharge. On physical examination, he had a temperature of 97.3°F, blood pressure of 110/65 mmHg, and heart rate of 75 beats per minute. He had a respiratory rate of 18 breaths per minute and an oxygen saturation of 97% on room air. There was purulent and foul smelling discharge from his right nostril, which was chronic according to our patient. During anterior rhinoscopy of the right nasal cavity, a hard foreign body coated with purulent secretions was found. There was minimal tenderness on percussion of the maxillary sinuses. Cardiac examination revealed an irregular rhythm with a grade 2/6 systolic murmur at the apex. He had minimal crackles bilaterally in his lower posterior chest. His abdomen was mildly distended and he had pitting edema in his lower extremities, while the rest of the physical examination was unremarkable.

**Figure 1:**
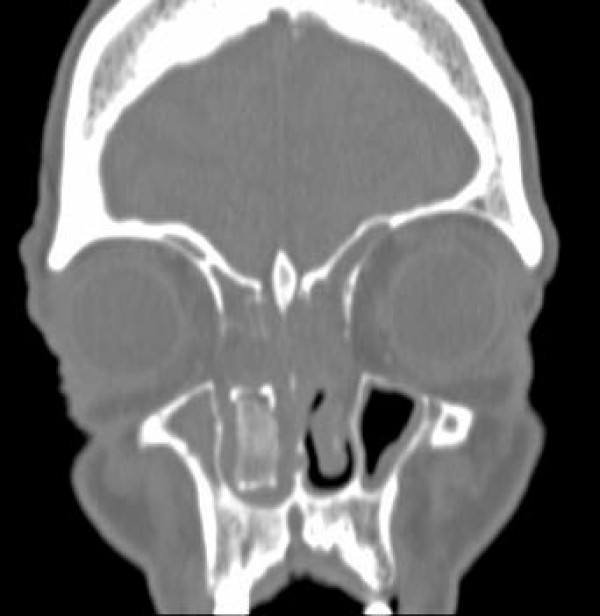
**Coronal view of the intra-nasal foreign body**.

**Figure 2:**
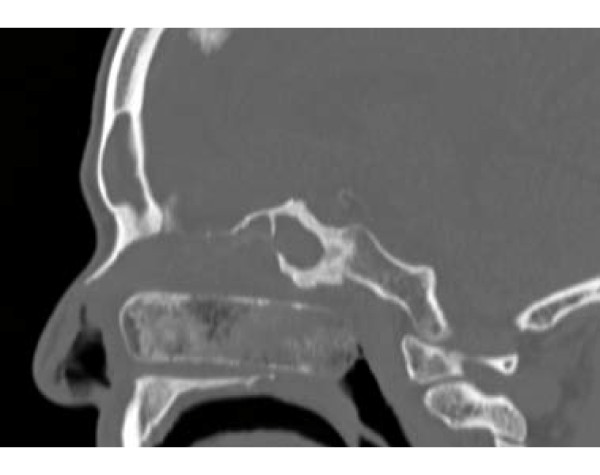
**Sagittal view of the calcified nasal packing**.

**Figure 3:**
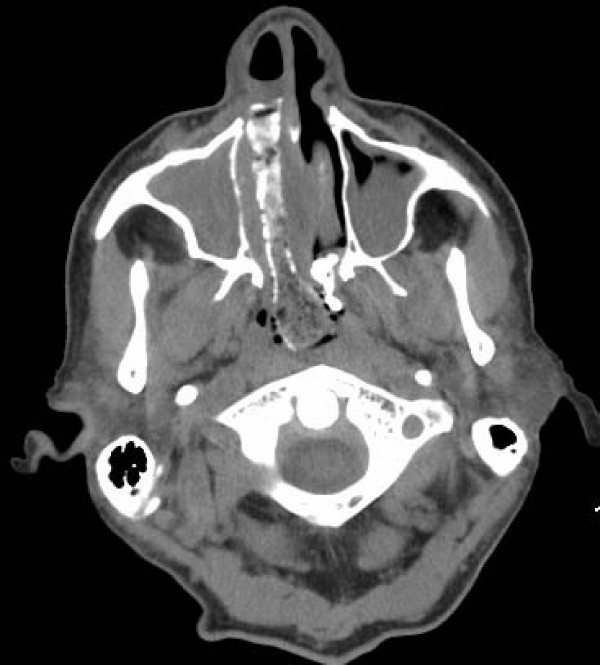
**Transverse view of the calcified foreign body**. Extensive sinusitis of the right and left maxillary sinuses is evident.

A complete blood count was unremarkable, showing a white blood cell count of 7.7 × 10^3^/mm^3 ^(74% neutrophils). A basic metabolic panel revealed chronic stable renal insufficiency with a serum creatinine of 1.7 mg/dl. A portable chest X-ray demonstrated marked cardiomegaly. A computed tomography scan of our patient's sinuses revealed the presence of extensive sinusitis of the right and left maxillary sinus and the presence of a calcified foreign body in the right nostril (Figures 1, 2, 3), leading to a diagnosis of bilateral sinusitis. On further investigation, our patient reported having had packing for the right nostril 12 years ago for a nosebleed. However, he was not sure if the packing had ever been removed and did not remember if the cause of his nosebleed was identified. The foreign body identified in the computed tomography represents the packing that had remained in the nostril all these years and became calcified with a bone like density at certain areas (Figures 1, 2, 3). The foreign body was quite large extending to the posterior nasopharynx and obliterating the drainage of the right maxillary sinus, causing extensive sinusitis.

However, multiple attempts to remove the foreign object with different techniques through the anterior nares, such as use of cupped forceps (including Tilley nasal forceps), hemostats, curved hooks, Fogarty biliary catheter, Howarth's periosteal elevator and suction [1] by ear, nose and throat (ENT) specialist, were unsuccessful. Our patient refused surgical removal of the foreign body. He also declined further antimicrobial treatment. He was discharged after diuresis and resolution of the leg edema.

## Discussion

A review of the literature shows that intra-nasal foreign bodies have been frequently reported especially among children. Among adults, however, they occur very rarely and are caused mostly by injury in an accident, trauma or coexisting mental disorders [2]. In a large study of 420 cases of foreign bodies in the nasal cavity only one adult case, a homeless man with nasal myiasis was described [3]. Unusual foreign bodies including buttons have been described very rarely in adults [4].

The majority of cases of intra-nasal foreign bodies are asymptomatic, except for a history of a foreign body having been inserted in the nose. Common symptoms, if present, include pain or discomfort, nasal discharge, nasal congestion or nasal odor. A unilateral mucopurulent nasal discharge with foul odor is the most consistent finding in patients with a nasal foreign body [1]. Rare symptoms have been reported, including bromidrosis (foul body odor) [5] and infections, such as facial cellulites [6], epiglottitis [7], and cephalic tetanus [8]. Differential diagnoses of a unilateral nasal obstruction include nasal polyp, nasal tumor, nasal abscess, septal hematoma, or unilateral choanal atresia [1].

Many foreign bodies are inert and can remain in the nose for years without mucosal damage. However, most foreign objects initiate congestion, swelling of the mucosa, ulceration, mucosal destruction and epistaxis. This can result in a foul fetor and rhinolith formation. Certain foreign bodies, such as vegetable, absorb water from the tissues and swell and can evoke an intense inflammatory reaction that can be sufficient to produce toxemia [9]. Thus, several important complications may occur with the presence of a nasal foreign body, including formation and development of rhinoliths, erosion into a contiguous structure, toxic shock syndrome and development of infections in surrounding structures including acute sinusitis or otitis media, periorbital cellulitis, meningitis, acute epiglottitis, diphtheria, and tetanus [7,8].

Long-standing objects left in body orifices tend to act as nuclei for concretion to form calculus deposits and become encrusted with calcified material and granulation tissue by receiving a coating of calcium, magnesium phosphate, and carbonate with time. Moreover, various iatrogenic foreign bodies on patients have been reported to cause nucleation and deposition of calculi [10]. Similarly, the nasal packing in our case had become calcified. Interestingly, there are reports of intra-nasal foreign objects that were left calcified *in situ *from two to 50 years [2,9,11]. Most nasal foreign bodies can be easily removed in the office or emergency department [1,9]. However, multiple attempts to remove the foreign object in our patient with different techniques [1] by ear nose and throat (ENT) specialist were unsuccessful.

This case is unusual and interesting for several reasons. To the best of our knowledge, the nasal packing in this case is the largest foreign body left *in situ *for over 10 years. The foreign body had essentially obliterated the whole right nasopharynx (Figures 1, 2, 3). Impressively, although our patient was diabetic and at an increased risk for development of complications from the foreign body and the chronic sinusitis including brain abscess, meningitis and toxic shock syndrome, the foreign body had not caused any symptoms for many years with the exception of nasal discharge the last few years. According to our patient, he neither had fever nor headache, and he did not pay attention to the nasal discharge. Our patient started having a nasal discharge the last few years that was likely attributable to the degradation of the foreign body. Degradation products might have produced local mucosal irritation and the production of excess mucus.

Although we could not determine the nature of the material of the nasal packing since it was not removed, it is possible that the nasal packing consisted of a relatively inert material that did not precipite significant mucosal damage or inflammatory reaction. Although specific defects in innate and adaptive immune function have been identified in diabetic patients, defects in adaptive immunity, which is important against foreign bodies, are less well-characterized [12]. Moreover, the link between glycemic control and the risk of common community-acquired infections including sinusitis is less established [12]. Extensive calcification of the foreign body in the setting of microangiopathy in patients with diabetes could also be a barrier for inflammatory response. Thus, the presence of an inert, heavily calcified material in an area with possible microangiopathy could potentially explain the absence of significant inflammatory response to this foreign object and its presence in the nasal cavity for so many years without any complications.

It is noted that our patient lives independently and he is not known to have a mental illness. Thus, our case, to the best of our knowledge, represents the first case in the literature of such a large intra-nasal foreign body described in an adult without mental illness and without trauma that remained *in situ *for such a long time.

## Conclusions

Undoubtedly, even illnesses that are not complicated could prove difficult for clinicians to diagnose. Clinicians should recognize the underlying causes that are responsible for symptoms of chronic sinusitis. This case emphasizes the importance of history-taking and a broad differential diagnosis. A unilateral nasal discharge should be assumed to be caused by an intra-nasal foreign body until proven otherwise.

## Competing interests

The authors declare that they have no competing interests.

## Consent

Written informed consent was obtained from our patient for publication of this case report and any accompanying images. A copy of the written consent is available for review by the Editor-in-Chief of this journal.

## Authors' contributions

TK analyzed and interpreted the data of our patient and was a major contributor in writing the manuscript. SO and HD analysed our patient data and contributed in writing the manuscript. All authors read and approved the final manuscript.
